# Magnetic ground state of FeSe

**DOI:** 10.1038/ncomms12182

**Published:** 2016-07-19

**Authors:** Qisi Wang, Yao Shen, Bingying Pan, Xiaowen Zhang, K. Ikeuchi, K. Iida, A. D. Christianson, H. C. Walker, D. T. Adroja, M. Abdel-Hafiez, Xiaojia Chen, D. A. Chareev, A. N. Vasiliev, Jun Zhao

**Affiliations:** 1 State Key Laboratory of Surface Physics and Department of Physics, Fudan University, Shanghai 200433, China; 2 Research Center for Neutron Science and Technology, Comprehensive Research Organization for Science and Society, Tokai, Ibaraki 319-1106, Japan; 3 Quantum Condensed Matter Division, Oak Ridge National Laboratory, Oak Ridge, Tennessee 37831, USA; 4 Department of Physics and Astronomy, University of Tennessee, Knoxville, Tennessee 37996, USA; 5 ISIS Facility, Rutherford Appleton Laboratory, STFC, Chilton, Didcot OX11 0QX, United Kingdom; 6 Center for High Pressure Science and Technology Advanced Research, Shanghai, 201203, China; 7 Faculty of Science, Department of Physics, Fayoum University, 63514 Fayoum, Egypt; 8 Institute of Experimental Mineralogy, Russian Academy of Sciences, 142432 Chernogolovka, Russia; 9 Institute of Physics and Technology, Ural Federal University, 620002 Ekaterinburg, Russia; 10 Low Temperature Physics and Superconductivity Department, M.V. Lomonosov Moscow State University, 119991 Moscow, Russia; 11 National University of Science and Technology ‘MISiS', Moscow 119049, Russia; 12 Collaborative Innovation Center of Advanced Microstructures, Nanjing 210093, China

## Abstract

Elucidating the nature of the magnetism of a high-temperature superconductor is crucial for establishing its pairing mechanism. The parent compounds of the cuprate and iron-pnictide superconductors exhibit Néel and stripe magnetic order, respectively. However, FeSe, the structurally simplest iron-based superconductor, shows nematic order (*T*_s_=90 K), but not magnetic order in the parent phase, and its magnetic ground state is intensely debated. Here we report inelastic neutron-scattering experiments that reveal both stripe and Néel spin fluctuations over a wide energy range at 110 K. On entering the nematic phase, a substantial amount of spectral weight is transferred from the Néel to the stripe spin fluctuations. Moreover, the total fluctuating magnetic moment of FeSe is ∼60% larger than that in the iron pnictide BaFe_2_As_2_. Our results suggest that FeSe is a novel *S*=1 nematic quantum-disordered paramagnet interpolating between the Néel and stripe magnetic instabilities.

## Abstract (web summary)

Different ground states of high-temperature superconductors reveal complex nature of magnetism. Here, Wang *et al*. report stripe and Néel spin fluctuations coexisting with non-magnetic nematic phase in FeSe, providing a viewpoint towards understanding the magnetism of cuprate and iron-based superconductors.

## Graphical abstract

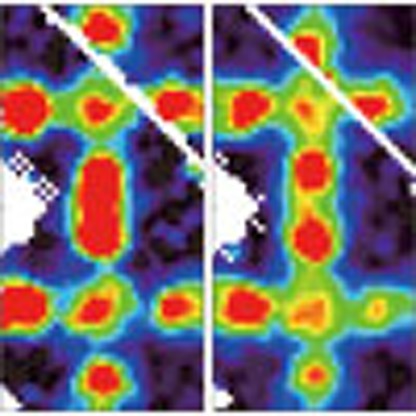

Recently, FeSe has attracted considerable interest because of its atypical magnetism^1^,^2^,^3^,^4^,^5^,^6^ and fascinating superconducting properties^7^,^8^,^9^,^10^. Although the superconducting transition temperature *T*_c_ of bulk FeSe (*T*_c_≈8 K) is low, it increases drastically under pressure (*T*_c_≈37 K; ref. 7), by carrier doping (*T*_c_≈40–48 K; refs 8, 9), or in the mono layer limit (*T*_c_∼65-109 K, ref. 10). The unique superconducting properties of FeSe are presumably related to its magnetism, which has also been shown to be uncommon. FeSe displays nematic, but not stripe magnetic order^1^,^11^ that is unexpected because nematic order has been argued to be the consequence of stripe magnetic order, and both break the C_4_ lattice symmetry^11^. In iron pnictides, the stripe magnetic order invariably occurs at or immediately below the nematic-(tetragonal-to-orthorhombic) ordering temperature^11^. Although previous works have shown that the nematic order could be driven by spin fluctuations without the requirement of magnetic order^2^,^3^,^4^,^12^, the microscopic origin of the absence of the long-range stripe magnetic order in FeSe remains elusive.

Theoretical studies have suggested that the stripe magnetic order in FeSe is absent due to the development of other competing instabilities^2^,^3^,^4^,^5^,^6^. Several ground states have been proposed, including Néel order^2^, staggered dimer/trimers/tetramers magnetic order^3^, pair-checkerboard order^5^, spin antiferroquadrupolar order^4^ and charge current-density wave order^6^. In experimental studies, neutron-scattering measurements showed substantial low-energy stripe spin fluctuations in single crystal^12^ and powder samples^13^. However, because of the limitations of the *q*-space information that can be obtained for powder measurements and the relatively narrow energy range probed previously, the precise nature of the magnetic ground state remains undetermined; this elucidation requires measurements of the momentum structure of the spin-fluctuation spectrum from low energy to the zone boundary over the entire Brillouin zone.

In this paper, we used inelastic neutron scattering to map out the spin-fluctuation spectra over the entire Brillouin zone in single-crystalline FeSe (Methods). Our data reveal the coexistence of the stripe and Néel spin fluctuations, both of which are coupled with nematicity. In addition, although the spin-fluctuation bandwidth is lower, the total fluctuating magnetic moment (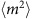
=5.19 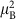/Fe) of FeSe is ∼60% larger than that in the iron pnictide BaFe_2_As_2_ (ref. 14). These findings suggest that FeSe is an *S*=1 nematic quantum-disordered paramagnet, interpolating between the Néel and stripe magnetic instabilities^2^.

## Results

### Momentum and energy dependence of spin fluctuations

**Figure 1:**
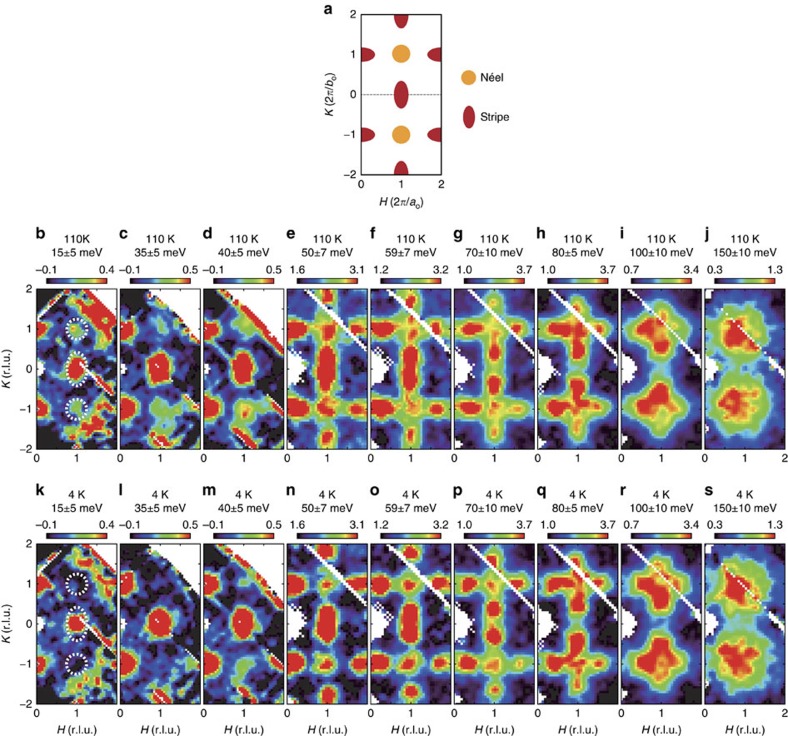
Momentum dependence of the spin fluctuations in FeSe at 4 and 110 K. (**a**) Schematic representation of the stripe and Néel spin fluctuations in the (*H*, *K*) plane. (**b**–**j**) Constant-energy images acquired at 110 K at indicated energies. (**k**–**s**) Constant-energy images obtained at 4 K at the same intensity scale as those acquired at 110 K. The measurements in (**b**–**d**,**k**–**m**) and (**e**–**j**,**n**–**s**) were carried out on ARCS with the incident neutron energy of 79 and 294 meV, respectively. The sample has two equally populated orthogonal twin domains in the *ab* plane at 4 K and the intensities near (1, 0) and (0, 1) are roughly the same. Symmetry equivalent data were pooled to enhance statistical accuracy. The |**Q**|-dependent background is subtracted for the data (**b**–**d**,**k**–**m**) below the aluminium phonon cutoff energy of ∼ 40 meV. Above 40 meV, raw data are presented (**e**–**j**,**n**–**s**). The colour bars indicate intensity in unit of mbar sr^−1^ meV^−1^ f.u.^−1^. The dashed ellipses and circles mark the stripe and Néel wavevectors, respectively.

Figure 1 shows the constant-energy images of spin fluctuations in the (*H*, *K*) plane. Here, in the high-temperature tetragonal phase (110 K), the spin response is strongest at **Q**=(1, 0) (marked by dashed ellipse) at 15 meV (Fig. 1b), which is consistent with previous low-energy measurements^12^,^13^. With an increase in energy (Fig. 1c–j), the spin fluctuations show anisotropic dispersion and counter-propagate mainly along the *K* direction. This is analogous to the stripe spin fluctuations detected in other iron-based superconductors^11^,^14^,^15^. Most notably, in addition to the stripe spin fluctuations, comparatively weaker, but clear scattering appears near (1, 1) (Fig. 1b, dashed circle), which implies the presence of spin fluctuations associated with the Néel magnetic instability (Methods); different from the anisotropic stripe spin fluctuations, the Néel spin fluctuations are nearly isotropic in the transverse and longitudinal directions (Fig. 1b–g). With an increase in the energy up to ∼150 meV, the Néel and stripe spin fluctuations overlap and cover a broad area centred at (1, 1) (Fig. 1j). On cooling to within the nematic phase (*T*=4 K), the Néel spin-fluctuation signal weakens considerably and is almost undetectable <35 meV (Fig. 1k,l). On the other hand, the momentum structure of the stripe spin fluctuation is essentially unchanged above and below *T*_s_ (Fig. 1k–s).

**Figure 2:**
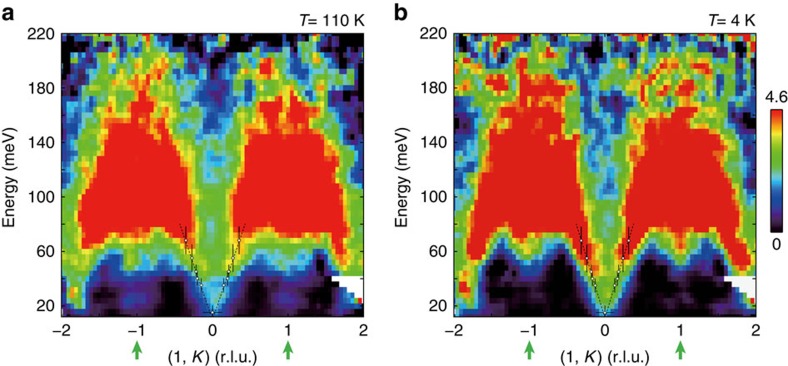
Dispersions of the stripe and Néel spin fluctuations in FeSe at 4 and 110 K. Background-subtracted *E*–*K* slice of the spin fluctuations at various incident energies: (**a**) *T*=110 K; (**b**) *T*=4 K. The data at *E*≥40 and *E*≤40 meV were collected on ARCS by using incident energy of 294 and 79 meV, respectively. The isotropic Fe^2+^ magnetic form factor is corrected for both sets of the data. The spectral weight transfer from the Néel (1, 1) to stripe (1, 0) wavevector below ∼70 meV on cooling to 4 K can be clearly seen. The open circles are dispersions obtained from the constant-energy cuts <68 meV in Fig. 3. The colour bar indicates intensity in unit of mbar sr^−1^ meV^−1^ f.u.^−1^. The vertical bars indicate the energy integration range. The horizontal bars at 15 meV indicate the full-width at half-maximum of the Gaussian fittings in Fig. 3i. The horizontal bars at other energies are the errors derived by least-square fittings. The dashed lines are a guide to the eye.

To further elucidate the spin fluctuations in *E*–**Q** space, we projected the spin fluctuations along the *K* direction near (1, 0) and (1, 1) (Fig. 2). Because the incident neutron beam was parallel to the *c* axis, the energy transfer was coupled with *L*. No *L* modulations were observed from the scattering near (1, 0, *L*) and (1, 1, *L*), which indicates a two-dimensional nature of the magnetism. As shown in Fig. 2a, the stripe spin fluctuations stem from (1, 0), split into two branches at ∼35 meV and extend up to above ∼150 meV at 110 K. The steeply dispersive Néel spin fluctuations are also visible (green arrowheads). As the temperature is lowered to 4 K, the Néel spin fluctuation exhibits a ∼30 meV gap, while the stripe spin fluctuations <70 meV are clearly enhanced (Fig. 2b).

**Figure 3:**
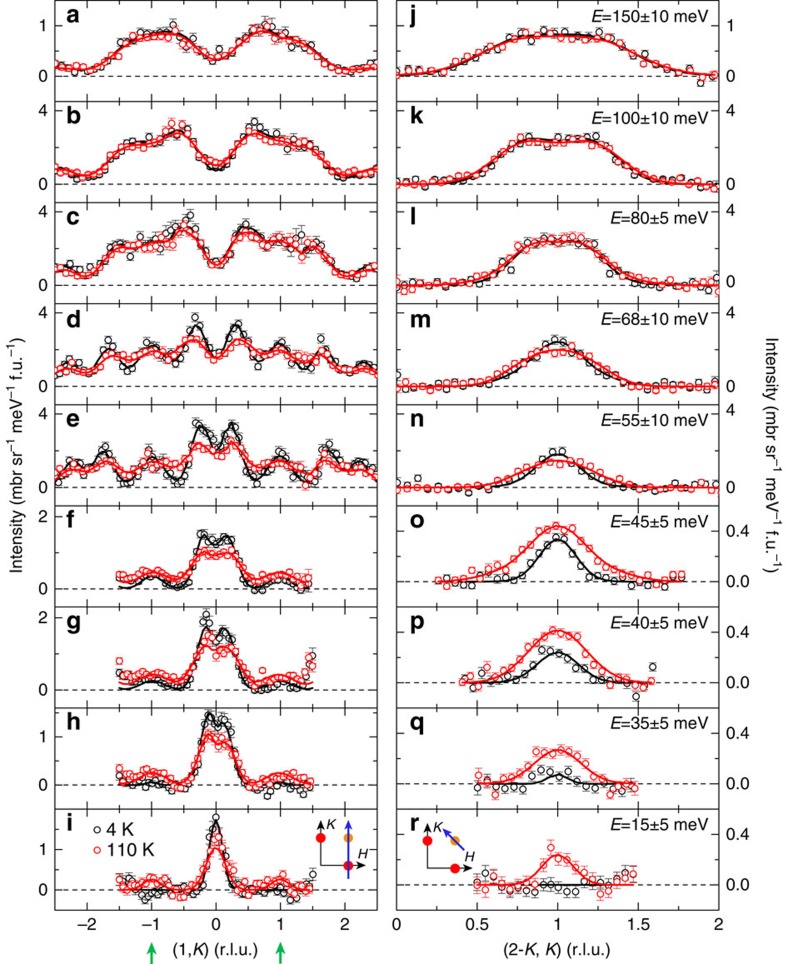
Constant-energy cuts of the stripe and Néel spin fluctuations in FeSe at 4 and 110 K. (**a**–**i**) Constant-energy cuts through the stripe and Néel magnetic wavevectors along the *K* direction at 4 and 110 K. (**j**–**r**) Constant-energy cuts through the Néel magnetic wavevector **Q**=(1, 1) along the transverse direction. The scan directions are marked by the arrows in the insets. The peak positions (dispersions) are determined by fitting with Gaussian profiles convoluted with the instrumental resolution, with the Fe^2+^ magnetic form factor corrected. The fitted dispersions are shown in Fig. 2. The error bars indicate 1 s.d.

To quantify the dispersions and intensities of the stripe and Néel spin fluctuations, we made constant-energy cuts at distinct energies (Fig. 3). As Fig. 3d–i show, at *T*=110 K, the single peak centred at (1, 0) at 15 meV evolves into a pair of peaks along the *K* direction at *E*≥35 meV. By contrast, the peak position of the Néel spin fluctuation (see green arrowheads) shows little change; it only broadens gradually in wavevector with increasing energy. The Néel spin fluctuation is more clearly visible along the transverse direction (Fig. 3m–r) because of the comparatively weaker influence of the stripe spin fluctuations. Here, double peaks formed due to the dispersion are not seen <68 meV, because the Néel spin fluctuations are commensurate and steeply dispersive (Fig. 3m–r). At higher energies, the Néel and stripe spin-fluctuation spectra merge with each other, and their dispersions cannot be determined unambiguously (Fig. 3a–c,j–l). The Néel spin fluctuation becomes featureless at low energies (Fig. 3i,r) at 4 K, which agrees with the results shown in the constant-energy and *E*–**Q** images (Figs 1k and 2b). We attempted to fit both types of spectra concurrently using a linear spin-wave theory for the two-neighbour (*J*_1(*a/b*)_*–J*_2_) or three-neighbour (*J*_1(*a/b*)_*–J*_2_*–J*_3_) Heisenberg model, where *J*_1(*a/b*)_, *J*_2_ and *J*_3_ are nearest neighbour (in the *a/b* direction), next-nearest neighbour and next next-nearest neighbour exchange coupling constants, respectively; but this was unsuccessful mainly because this theory cannot account for the observed strong low-energy spin excitations at both (1, 0) and (1, 1).

### Momentum integrated local susceptibility above and below *T*_s_

**Figure 4:**
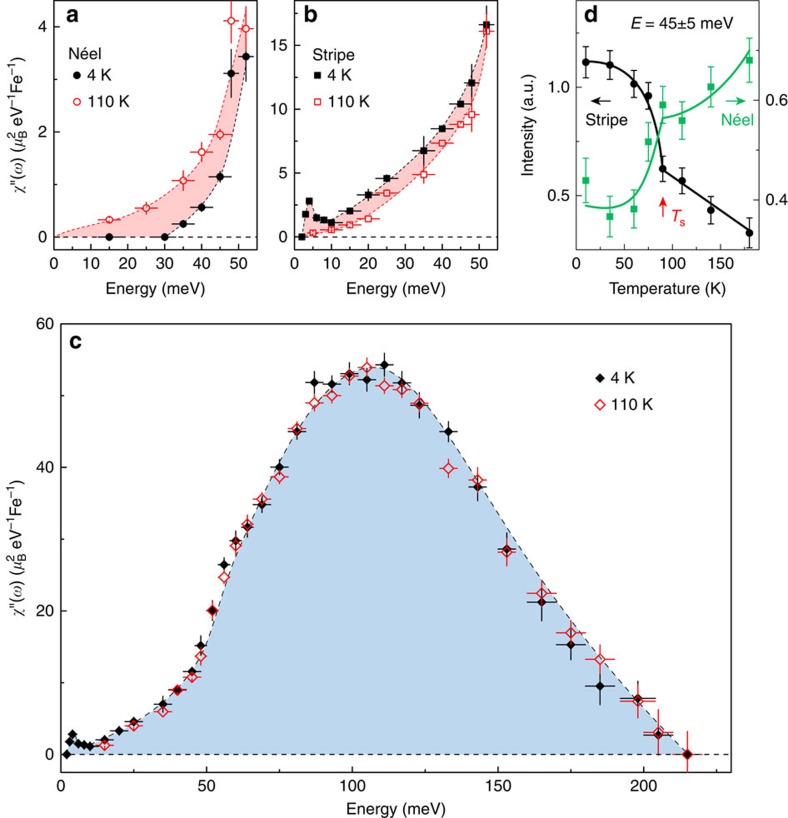
Energy dependence of the local susceptibility *χ*″(*ω*) for FeSe at 4 and 110 K. (**a**–**c**) Energy dependence of *χ*″(*ω*), at 4 and 110 K, calculated for (**a**) the Néel spin fluctuations, (**b**) the stripe spin fluctuations, and (**c**) the sum of the stripe and Néel spin fluctuations. A resonance mode is clearly observed at ∼4 meV and 4 K, whose intensity is consistent with the previous low-energy measurements normalized with acoustic phonons^12^. (**d**) Temperature dependence of the intensities of the stripe and Néel spin fluctuations. The data in **a**–**c** were collected on ARCS (incident energy *E*_i_=294, 79, 40 meV) and 4SEASONS (*E*_i_=21, 13.6 meV). The data in **d** were collected on MERLIN (*E*_i_=123.4 meV). The horizontal and vertical bars indicate the energy integration range, and the statistical errors of 1 s.d., respectively. The solid and dashed lines are a guide to the eye.

More insight into the nature of the underlying magnetic ground state and its interaction with the nematicity could be acquired by calculating in absolute units the energy dependence of the momentum integrated local susceptibility *χ*″(*ω*) above and below *T*_s_; as Fig. 4a,b show, at *T*=110 K, the Néel spin-fluctuation spectral weight is roughly 26% of that of the stripe spin fluctuation <52 meV, where the two signals are well separated in *q*-space. Upon cooling to *T*=4 K, the spectral weight loss for the Néel spin fluctuations is approximately recovered by the enhanced stripe spin fluctuations (red shaded areas), and thus the total local susceptibility *χ*″(*ω*) does not show a marked change across *T*_s_ (Fig. 4c). Moreover, the detailed temperature dependence of the stripe and Néel spin fluctuations show that the spectral weight transfer is clearly coupled with the development of the nematic phase (Fig. 4d). At both 4 and 110 K, the total *χ*″(*ω*) exhibits a high maximum at ∼105 meV and extends up to 220 meV (Figs 2a,b and 4c). This bandwidth is considerably lower than that (∼340 meV) of the stripe ordered BaFe_2_As_2_ (ref. 14), which is very likely due to the competition between the Néel and stripe magnetic instabilities. Clearly, this type of competition also prevents the long-range magnetic order in FeSe. By integrating the spectral weight from low energy to the zone boundary, we determined that the total fluctuating moment at 4 and 110 K are 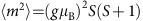
**=**5.19±0.32 and 5.12±0.27 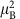/Fe, respectively, which are larger than those in the superconducting BaFe_1.9_Ni_0.1_As_2_ (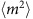
=3.2 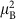/Fe) and stripe-ordered BaFe_2_As_2_ (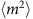
=3.17 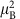/Fe) (ref. 14). Accordingly, this yields an effective spin of *S*≈0.74 in FeSe, which likely corresponds to an *S*=1 ground state in the presence of itinerant electrons.

## Discussion

The coexistence of the Néel and stripe spin fluctuations is unexpected because FeSe contains only one type of magnetic ions. This differs from the Mn-doped nonsuperconducting iron-pnictide compound BaFe_2−*x*_Mn_*x*_As_2_, where the Néel magnetic correlation is induced by the local moments of Mn, while the stripe magnetic correlation is induced by Fe, given that pure BaMn_2_As_2_ is a local-moment Néel type antiferromagnet^16^. Although density functional theory and dynamical mean-field theory failed to reproduce the observed band structure of FeSe (refs 17, 18), a random-phase approximation calculation with an engineered tight-binding band structure^19^ predicted the spin fluctuations near (1, 0) and (1, *q*). However, in this phenomenological model^19^, the (1, *q*) spin fluctuation is incommensurate and gapless below *T*_s_, which is inconsistent with our data. The relatively small spin-fluctuation bandwidth and large fluctuating moment together with the low-carrier density^17^,^18^ indicate that the magnetic moments in FeSe are more localized than in iron pnictides.

In the more localized case, we can exclude the previously proposed competing staggered dimer/trimers/tetramers magnetic order^3^ and pair-checkerboard order^5^. Here, the competition between the Néel and stripe magnetic instabilities could be instead understood within the framework of a frustrated *J*_1_*–J*_2_ model. In this model, for an *S*=1 system, the Néel order is stable for *J*_2_*/J*_1_≲0.525, whereas the stripe order is the ground state for 0.555≲*J*_2_*/J*_1_ (refs 20, 21). It was predicted that FeSe would be an *S*=1 nematic quantum paramagnet in the intermediate coupling region (0.525≲*J*_2_*/J*_1_≲0.555), which is characterized by gapped stripe and Néel spin fluctuations^2^,^21^. This agrees with our data that the majority of the spectral weight is concentrated at relatively high energies (∼100 meV) even in the presence of itinerant electrons (Figs 2a,b and 4c). Furthermore, in this scenario, the nematic order is viewed as a vestigial order that is retained when the static stripe order is suppressed by quantum fluctuations^2^. The finding that the stripe spin fluctuation carries considerably more spectral weight than the Néel spin fluctuation suggests that the system is indeed closer to stripe rather than to Néel order. On this basis, the temperature evolution of the spin fluctuations that we observed can be explained. As the stripe magnetic order breaks the C_4_ lattice symmetry, while the Néel order preserves it, the orthorhombic-/nematic-phase transition might partially lift the magnetic frustration and drive the system towards the stripe-ordered phase. Thus, the stripe spin fluctuations are enhanced, while the Néel spin fluctuations are suppressed and gapped in the nematic phase. These considerations lead to a natural understanding of the paramagnetic nematic phase in FeSe.

We now discuss the evolution of the magnetism, nematicity and superconductivity in FeSe, and its derivatives. Since the Néel spin fluctuation is gapped in the nematic phase, it is unlikely responsible for the electron pairing in bulk FeSe at ambient pressure. However, the competition between the Néel and stripe magnetic instabilities across *T*_s_ suggests that the magnetic ground state and superconductivity could be highly tunable. Indeed, it has been shown that high pressure not only enhances superconductivity, but also induces static magnetic order in FeSe (refs 22, 23). This is surprising as superconductivity always competes with the static magnetic order in iron pnictides. Further neutron diffraction measurements under pressure are required to clarify the nature of this magnetic order. In addition, the proximity of the Néel magnetic instability of FeSe might also have implications for our understanding of the magnetism in the K dosed^9^,^24^, molecule intercalated^25^ and mono layer^10^,^26^ FeSe, because in these heavily electron-doped compounds, the nematic order and the hole pockets are absent, and the electron pockets at two adjacent zone edges are connected by the Néel wavevector **Q**=(1, 1). Interestingly, this would be in analogy with the cuprate superconductors in terms of the magnetism and Fermi surface topology^27^. To further elucidate the role of the Néel spin fluctuations in iron-based superconductivity, a detailed study of the pressure-/electron-doping dependence of the spin correlations in FeSe would be desirable.

To conclude, we have reported the observation of the Néel spin fluctuations over a wide energy range in an iron-based superconductor. We show that the absence of the long-range magnetic order in FeSe is due to the competition between the Néel and stripe magnetic instabilities. This differs from the parent compounds of the cuprate and iron pnictide high-temperature superconductors, where only one type of magnetic order is observed. Our findings agree with a theoretical prediction that FeSe is a novel *S*=1 nematic quantum-disordered paramagnet interpolating between the Néel and stripe magnetic instabilities^2^, which indicates a connection between the magnetism of the cuprate- and iron-based superconductors. The experimental determination of the nematic magnetic ground state of FeSe will be extremely valuable in identifying the microscopic mechanism of superconductivity in FeSe-based materials^8^,^9^,^10^,^24^,^25^,^26^.

## Methods

### Sample growth and characterizations

Our FeSe single crystals were grown under a permanent gradient of temperature (∼400–330 °C) in the KCl–AlCl_3_ flux^28^. The single-crystal X-ray and neutron diffraction refinements on our samples indicated a stoichiometric chemical composition to within the error bars, and no interstitial atoms or impurity phases were observed (Supplementary Note 1; Supplementary Figs 1–3; Supplementary Table 1). The specific heat, magnetic susceptibility and resistivity measurements performed on randomly selected FeSe single crystals further demonstrate that our sample is a bulk superconductor without detectable impurities. (Supplementary Note 1; Supplementary Figs 4 and 5).

### Neutron-scattering experiments

Our inelastic neutron-scattering measurements were carried out on the ARCS time-of-flight chopper spectrometer at the Spallation Neutron Source of Oak Ridge National Laboratory, USA, 4SEASONS chopper spectrometer at the Japan Proton Accelerator Research Complex and MERLIN chopper spectrometer at the Rutherford Appleton Laboratory, Didcot, UK. The large detector arrays on these instruments allowed us to measure spin excitations over a wide range of energy and momentum. The |**Q**|-dependent background is subtracted for the data below the aluminium phonon cutoff energy of ∼40 meV (Supplementary Note 2; Supplementary Fig. 7). To facilitate comparison with theory and previous measurements, our data were normalized into absolute units by using the elastic incoherent scattering of a standard vanadium sample. The absolute intensity of the resonance mode is consistent with previous low-energy data^12^ normalized with acoustic phonons (Supplementary Fig. 6). The incident neutron beam was aligned parallel to the *c* axis. The wavevector **Q** at (*q*_*x*_*, q*_*y*_*, q*_*z*_) is defined as (*H, K, L*)=(*q*_*x*_*a*/2*π*, *q*_*y*_*b*/2*π*, *q*_*z*_*c*/2*π*) in the reciprocal lattice units in the orthorhombic unit cell. In this unit cell, the magnetic wavevectors associated with the stripe and Néel magnetic order are **Q**=(1, 0) and **Q**=(1, 1), which correspond to the ordering wavevectors of the parent compounds of the iron pnictides and the cuprates, respectively.

### Data availability

All relevant data that support the findings of this study are available from the corresponding author on request.

## Additional information

**How to cite this article**: Wang, Q. *et al*. Magnetic ground state of FeSe. *Nat. Commun.* 7:12182 doi: 10.1038/ncomms12182 (2016).

## Supplementary Material

Supplementary Information Supplementary Figures 1-7, Supplementary Table 1, Supplementary Notes 1-2 and Supplementary References
