# Supplementary material for: Magnetic ground state of FeSe
Source: Nat Commun. 2016 Jul 19;7:12182. doi: 10.1038/ncomms12182 (PMC4960298; doi:10.1038/ncomms12182)
Supplement: Supplementary Information — Supplementary Figures 1-7, Supplementary Table 1, Supplementary Notes 1-2 and Supplementary References [file ncomms12182-s1.pdf]

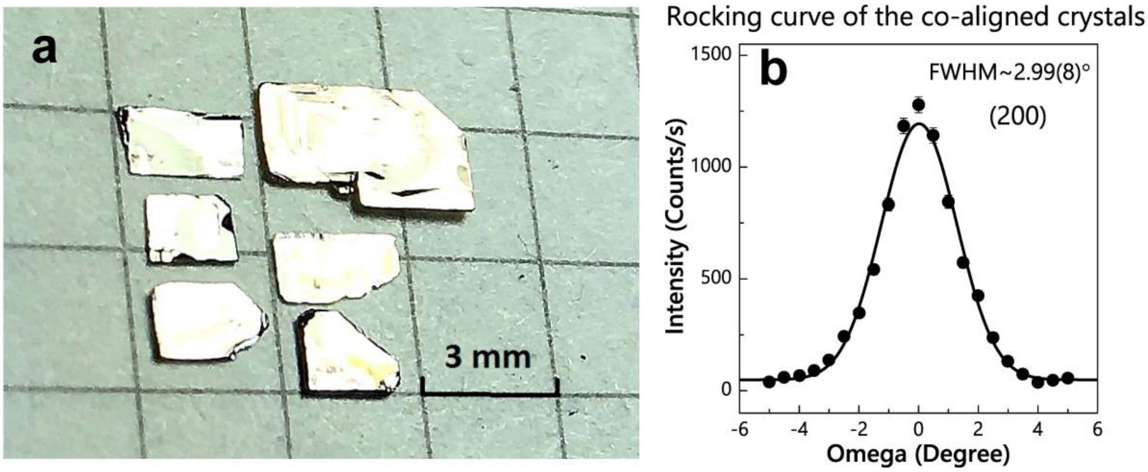

Supplementary Figure 1: Photograph of our FeSe single crystals and the rocking curve of the co-aligned crystals. (**a**) Photograph of representative FeSe single crystals (~2-20 mg each) used for inelastic neutron scattering (INS) measurements. The single crystals are plate-like with natural cleaved edges along the tetragonal a/b direction. The well-shaped habitus with metallic luster indicates the high quality of the crystal. (**b**) Rocking curve of the (200) Bragg peak of the co-aligned crystals used for INS measurements. The full width at half maximum (FWHM) of the rocking curve is 2.99(8)°. The error bars indicate one standard deviation.

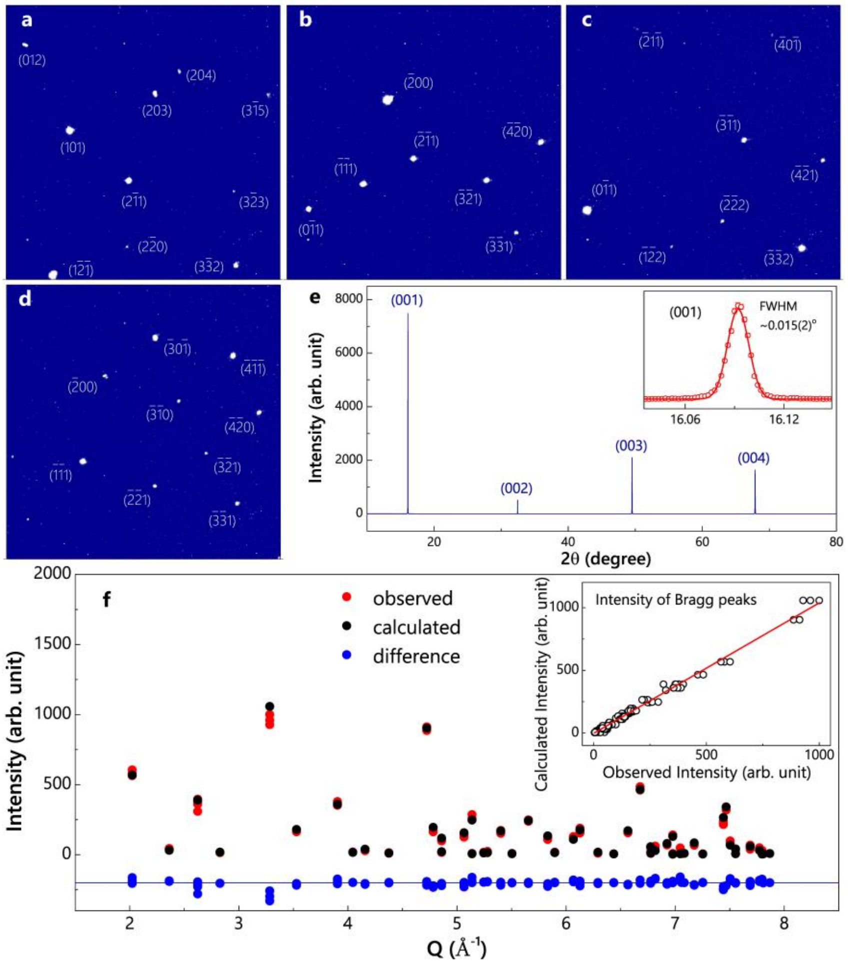

Supplementary Figure 2: Single crystal X-ray diffraction data and the Rietveld refinement results in FeSe. (**a-d**) Representative diffraction pattern shown on the area detectors at 298 K. The sharp bright spots are indexed as Bragg reflections of the tetragonal phase of FeSe. The measurements were performed on a single crystal from the same batch as those used for INS experiments. (**e**) The scans perpendicular to the cleaved surface (*ab* plane) show only (00L) Bragg peaks of the tetragonal FeSe. The FWHM of the (001) Bragg peak is ~ 0.015(2)°, indicating a good quality of the sample. (**f**) X-ray single crystal diffraction refinement results. The refined parameters are summarized in Table I of the supplementary information of ref. 2. Data are presented in the tetragonal notation. Our Rietveld refinements are based on 178 X-ray Bragg peaks using the FULLPROF program.

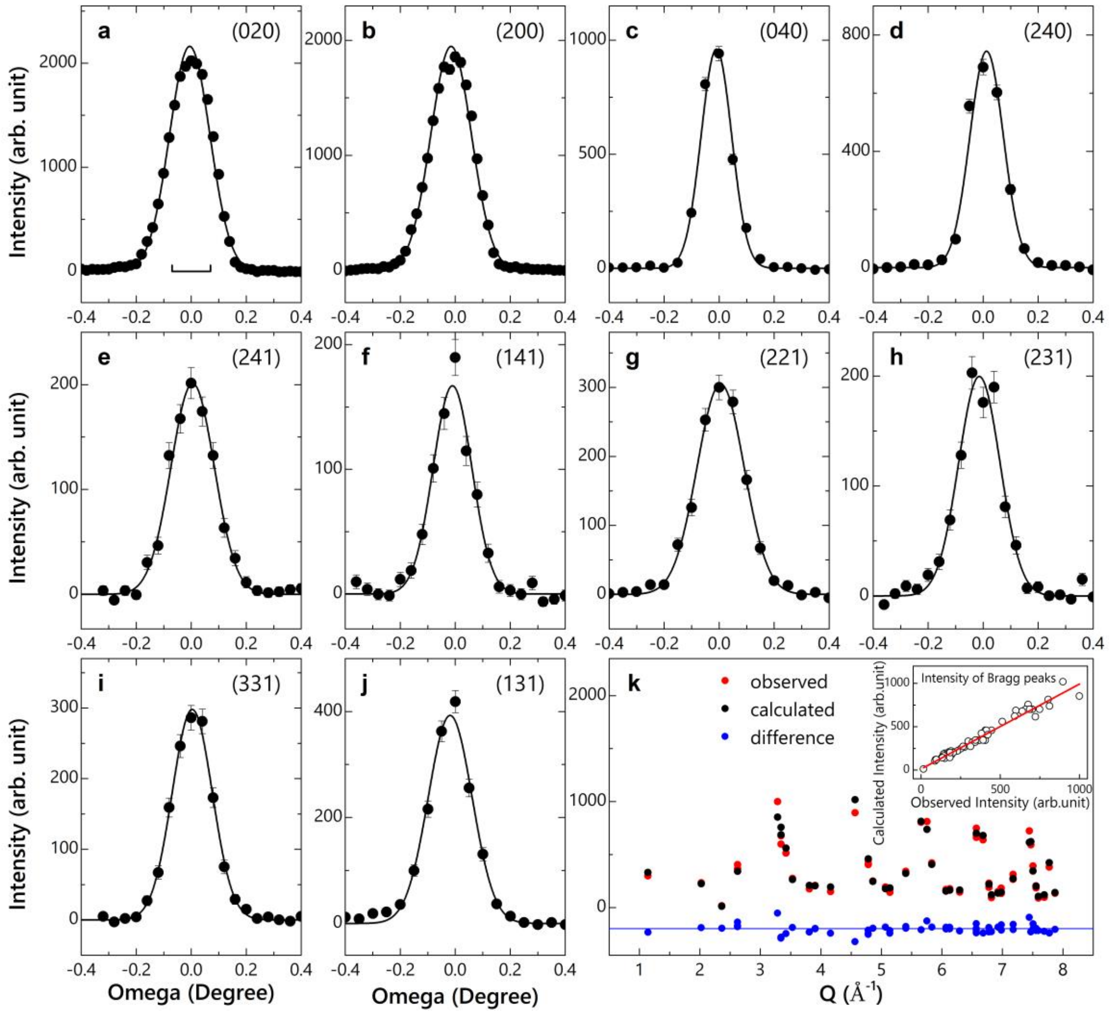

Supplementary Figure 3: Single crystal neutron diffraction data and the Rietveld refinement results in FeSe. (**a**-**j**) Representative neutron diffraction pattern measured at 296 K. The FWHM of the rocking curve of (020) peak is 0.173(3)°, which is essentially resolution limited. The horizontal bar indicates the instrument resolution. The measurements were performed on the HB3A four-circle single-crystal diffractometer at the High-Flux Isotope Reactor at the Oak Ridge National Laboratory. (**k**) Single crystal neutron diffraction refinement results. The refined parameters are summarized in Supplementary Table 1. Data are presented in the tetragonal notation. The Rietveld refinements are based on 71 neutron Bragg peaks using the FULLPROF program. The error bars indicate one standard deviation.

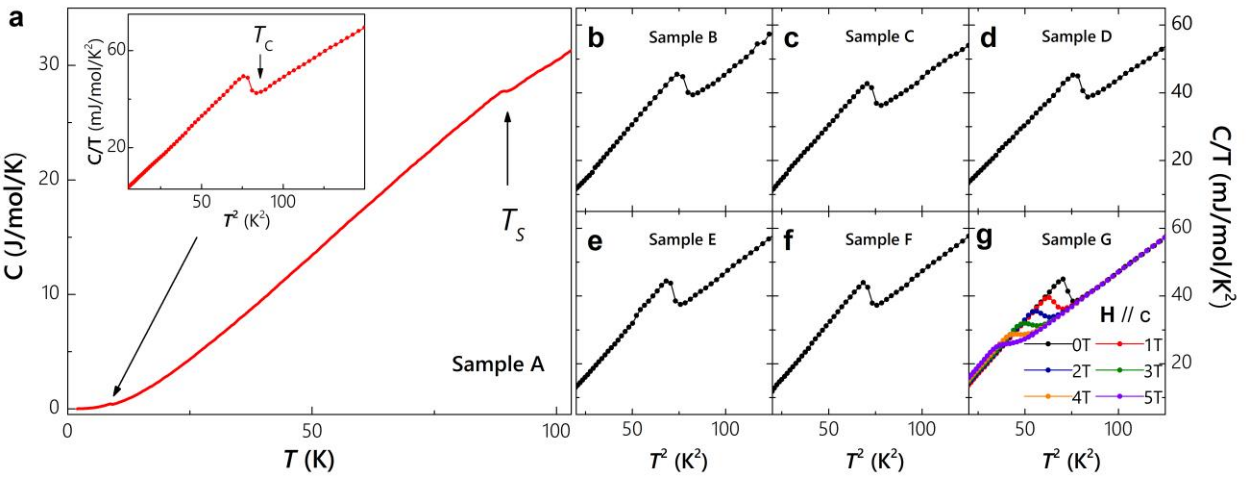

Supplementary Figure 4: Specific heat of FeSe single crystals. Samples A-G are randomly selected from the same batch as those used for the INS experiments. (**a**) Temperature dependence of specific heat of sample A. The superconducting and structural transitions are clearly observed. The inset shows the specific heat of sample A near T_c. (**b**-**f**) Low temperature specific heat of samples B-F. Sharp superconducting specific heat anomaly is observed in all samples. (**g**) Magnetic field dependence of the specific heat of sample G. The superconducting specific heat anomaly is progressively suppressed under magnetic field along the *c*-axis.

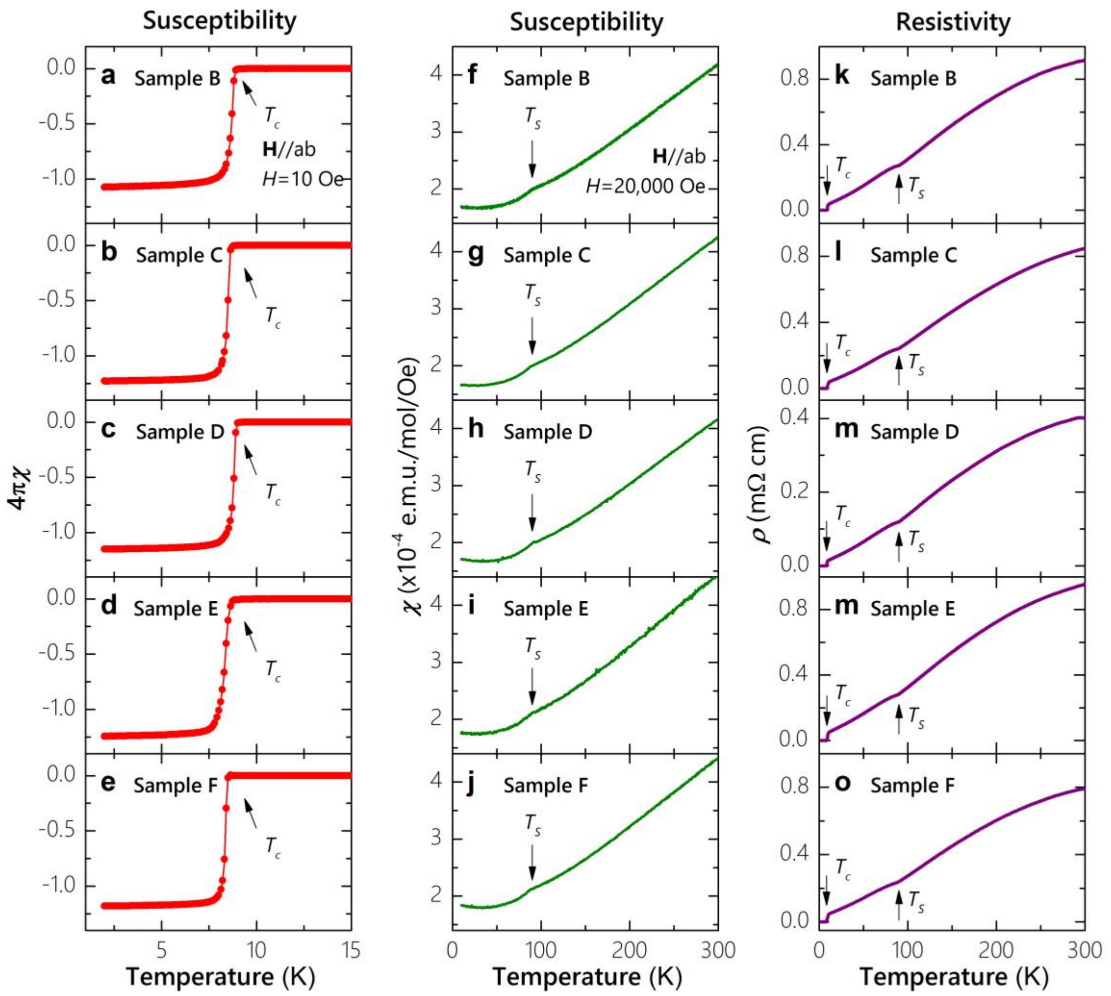

Supplementary Figure 5: DC magnetic susceptibility and resistivity of FeSe single crystals. Data were measured on the same samples used for the specific heat measurements. (**a-e**) Zero-field-cooled (ZFC) magnetic susceptibility measured in a magnetic field of H=10 Oe. A sharp superconducting transition is observed at ~8.7 K. The superconducting volume fraction is close to ~100% for all samples. The diamagnetic screening is slightly larger than -1 because of the demagnetization effect. (**f-j**) Magnetic susceptibility under a magnetic field of H=20000 Oe. The kink at 90 K corresponds to the structural (nematic) phase transition. (**k-o**) Temperature dependence of resistivity. The superconducting and structural transitions are clearly observed.

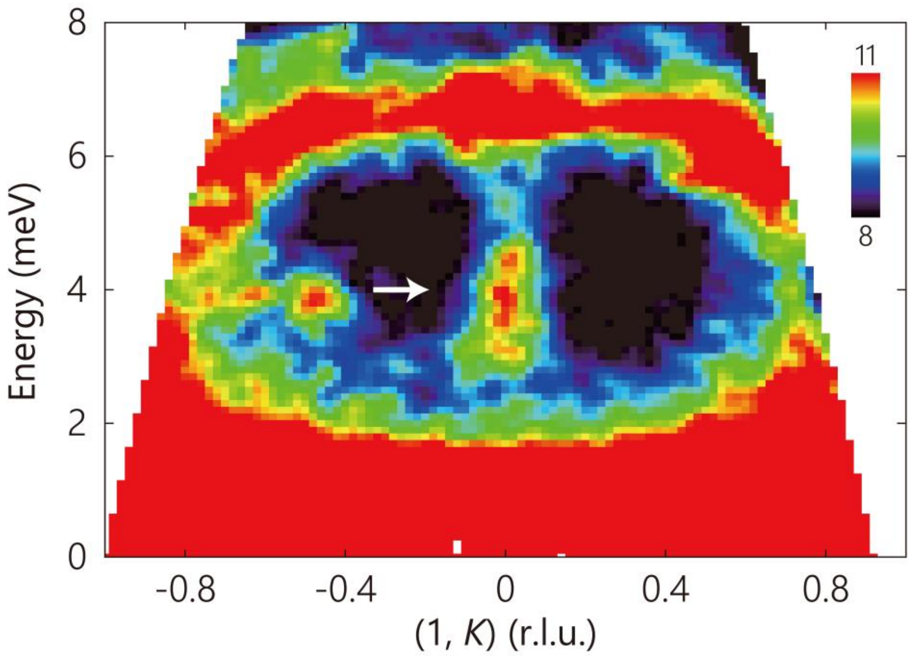

Supplementary Figure 6: Magnetic resonance mode. The E - K slice of the spin fluctuations was measured with E_i = 13.6 meV at 4 K on 4SEASONS (ref. 3). A magnetic resonance mode emerges at the stripe AFM wavevector **Q** = (1, 0) and $E \approx 4$ meV in the superconducting state (white arrow), which is consistent with our previous data measured on a triple axis spectrometer[2]. By contrast, the Néel spin fluctuation is gapped (up to ~ 30 meV) without a resonance mode at 4 K. The colour bar indicates intensity in arbitrary unit.

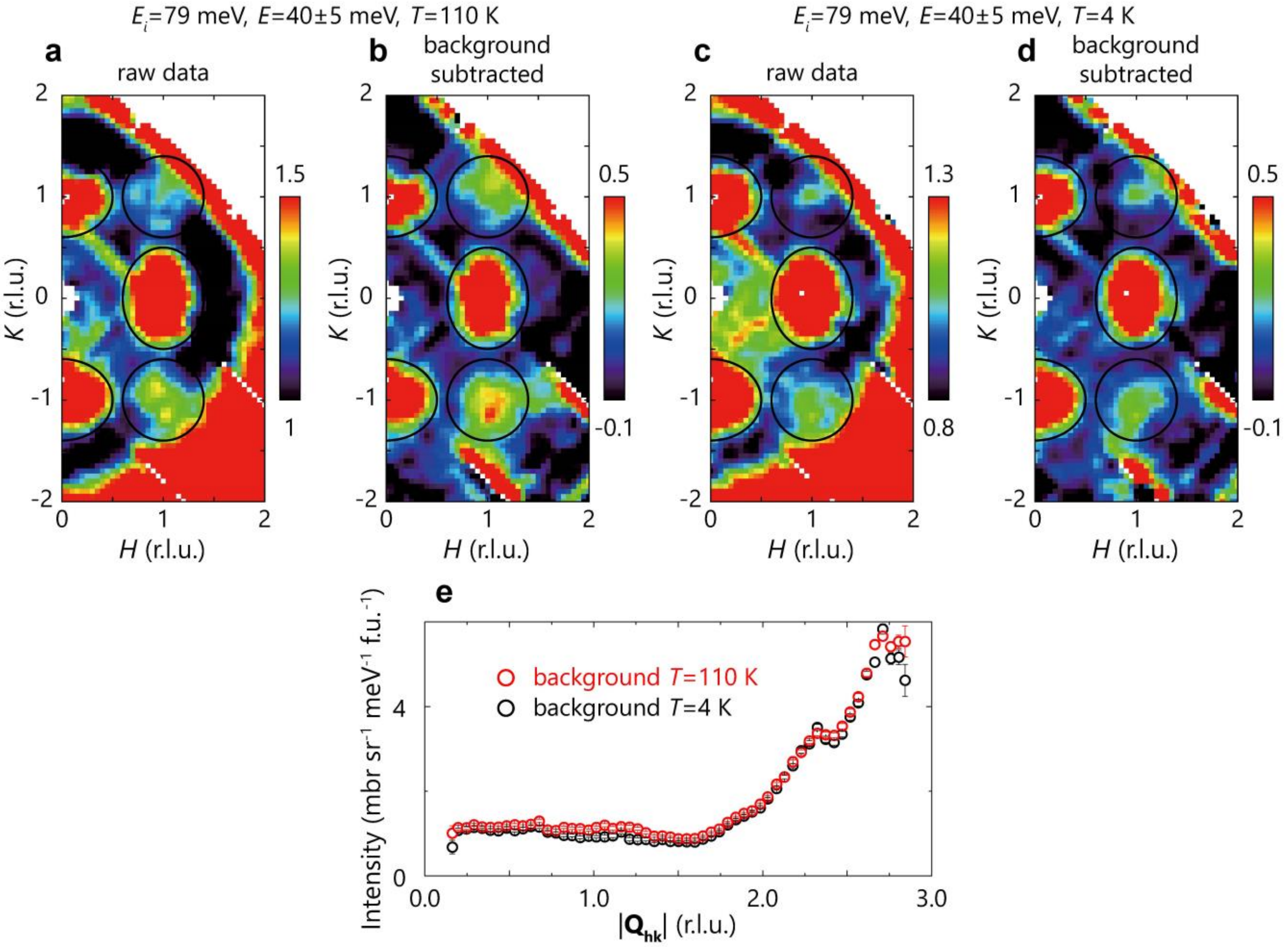

Supplementary Figure 7: Raw and background-subtracted constant energy images. (**a, b**) Raw (**a**) and background subtracted (**b**) constant energy images at 40 meV at 110 K. (**c, d**) Raw (**c**) and background subtracted (**d**) constant energy images at 40 meV at 4 K. The black ellipses indicate the magnetic signals. The ring-like phonon background from the aluminium sample holder/environment can be clearly seen in the raw constant energy images (red area at high |**Q**|). The red streaks are due to gaps between neutron detectors. (**e**) The |**Q**|-dependent background estimated from the scattering away from the magnetic signals. The colour bars indicates intensity in unit of mbar sr^{-1} meV^{-1} f.u.$^{-1}$. The error bars indicate one standard deviation.

Supplementary Table 1. Refined structure parameters and chemical composition of FeSe against neutron diffraction data at 296 K. Space group: *P*4/nmm (No. 129). Atomic positions: Fe: 2a (0, 0, 0); Se: 2c (0, 0.5, z).

	Refined composition	$FeSe_{0.99(3)}$
	a (Å)	3.7613(14)
	c (Å)	5.506(5)
Fe atom	B_{iso} (Å^2)	0.64(7)
Se atom	*z*	0.2660(16)
	B_{iso} (Å^2)	0.95(2)
	R_1	0.0492
	wRF^2	0.118
	χ^2	1.14

Supplementary Note 1: Sample characterizations

To determine the chemical composition and structural parameters of our FeSe single crystals, we have carried out single crystal X-ray and neutron diffraction measurements on single crystals from the same batch as those used for INS experiments. Supplementary Figure 2 shows the single crystal X-ray diffraction data and the Rietveld refinement results. The Rietveld refinements were performed using the FULLPROF program[1]. The refined results are summarized in Table I of the supplementary information of ref. 2. The Bragg reflections are clear and sharp (Supplementary Figs 2a-e), indicating a good crystallization quality, and no scattering from impurity phases or interstitial atoms were observed. The refined chemical composition is $FeSe_{0.990(10)}$, (i.e. stoichiometric within the error bars).

Supplementary Figure 3 presents the representative neutron diffraction patterns and the refinement results. The mosaic of the single crystal determined by the neutron rocking scan of the (020) peak is 0.173(3)°, which is essentially resolution limited (Supplementary Fig. 3a). The neutron diffraction refined chemical composition is $FeSe_{0.99(3)}$ (Supplementary Fig. 3k and Supplementary Table 1), which is consistent with the X-ray diffraction results.

We have performed specific heat, magnetization and resistivity measurements on randomly selected single crystals from the same batch as those used for INS experiments (Supplementary Figs 4 and 5). As Supplementary Figure 4 shows, the specific heat of our crystal exhibits a sharp jump near T_c. The magnetization and resistivity measurements give a very sharp superconducting transition at $T_c \sim 8.7$ K with a transition width of ~ 0.3 K, and the superconducting volume fraction is close to ~100% for all samples (Supplementary Figs 5a-e). We also note that the temperature dependence of the magnetic susceptibility and specific heat on our sample displays no indication of magnetic impurities.

Supplementary Note 2: Background subtraction

We used an aluminium sample holder/environment for our inelastic neutron scattering measurements because aluminium has a high transparency for neutrons. The phonon background from the polycrystalline aluminium sample holder/environment below its phonon cut-off energy of ~ 40 meV only depends on the amplitude of **Q** (similar to a powder ring), which could be easily subtracted. Supplementary Figure 7a and 7c show the raw data of constant energy images at E = 40±5 meV at 110 K and 4 K, respectively. A |**Q**|-dependent background was subtracted and the resultant data was shown in Supplementary Figure 7b and 7d in which the stripe and Néel magnetic signals can be seen more clearly. The |**Q**|-dependent background was estimated by the scattering away from the magnetic signals (black ellipses). For the data at E > 40 meV, no ring-like phonon background was observed and the raw constant energy images were presented in Fig. 1e-j, 1n-s.

Supplementary References

[1] Rodrguez-Carvajal, J. Recent advances in magnetic structure determination by neutron powder diffraction. *Physica (Amsterdan)*, **192B,** 55 (1993).

[2] Wang, Q. *et al.* Strong interplay between stripe spin fluctuations, nematicity and superconductivity in FeSe. *Nat. Mater.* **15,** 159-163 (2016).

[3] Kajimoto, R. *et al.* The Fermi Chopper Spectrometer 4SEASONS at J-PARC. *J. Phys. Soc. Japan* **80,** SB025 (2011).
